# Self-Related Cognitive Schemas, Negative Self-View, and Perceived Psychosocial Burden in Adults with Celiac Disease: A Cross-Sectional Comparative Study

**DOI:** 10.3390/nu18203457

**Published:** 2026-10-21

**Authors:** Kinga Kot, Ola Sobieska-Poszwa, Agnieszka Dobrowolska, Iwona Krela-Kaźmierczak, Ewa Misterska

**Affiliations:** 1 Poznań Institute of Psychology and Pedagogy, Varsovia University of Business & Applied Sciences, 02-304 Warszawa, Poland; kinga.aleksandra.kot@gmail.com; 2 Department of Pediatrics, Allergology and Gastroenterology, Ludwik Rydygier Collegium Medicum in Bydgoszcz, Nicolaus Copernicus University in Torun, 87-100 Toruń, Poland; ola.sobieska-poszwa@cm.umk.pl; 3 Poznan University of Medical Sciences, Poland, Department and Clinic of Gastroenterology, Dietetics and Internal Diseases, 60-512 Poznań, Poland; agdob@ump.edu.pl (A.D.); ikrela-kazmierczak@ump.edu.pl (I.K.-K.); 4 Department of Mental Health, Poznan University of Medical Sciences, 60-512 Poznań, Poland

**Keywords:** celiac disease, gluten-free-diet, cognitive schemas, depression

## Abstract

**Background**: Self-related cognitive schemas may influence psychological adaptation to chronic illness. However, their role in adults with celiac disease (CD) remains poorly understood. We aimed to assess cognitive schemas and depressive symptoms in adults with CD compared with healthy controls, and to examine their associations with perceived disease burden. **Methods**: This cross-sectional comparative study included 96 adults with CD and 96 healthy controls. Participants completed the Cognitive Triad Inventory (CTI) and Beck Depression Inventory (BDI), and sociodemographic and clinical data were collected. Perceived disease- and diet-related burden was assessed using investigator-developed single-item measures. **Results**: The mean age at CD diagnosis was 33.6 (SD 13.4) years, and the mean duration of symptoms before diagnosis was 5.4 (SD 5.1) years. Adults with CD reported higher depressive symptom scores than healthy controls (8.4 (SD 7.9) vs. 6.6 (SD 8.0); Mann–Whitney U = 3850.5, Z = 1.99, Cohen’s d = 0.29, *p* = 0.046). No significant between-group differences were observed in total CTI scores or its Self-view, World-view, or Future-view subscales. After adjustment for age, sex, educational level, marital status, and employment status, neither BDI nor CTI scores were independently associated with group membership. Exploratory bivariate analyses within the CD group showed that higher Self-view scores were associated with lower perceived negative impact of gluten-free diet requirements on family life (rS = −0.29, *p* = 0.004) and lower perceived impact of CD symptoms and disease exacerbations on everyday life (rS = −0.31, *p* = 0.002). These associations remained statistically significant after Bonferroni correction. **Conclusions**: Adults with CD reported slightly higher depressive symptom levels than healthy controls, whereas cognitive schemas did not differ significantly between groups. The observed associations between CTI Self-view and selected indicators of perceived disease- and diet-related burden warrant further investigation, but do not establish a causal contribution of self-view to disease burden.

## 1. Introduction

Celiac disease (CD) is a chronic immune-mediated enteropathy triggered by gluten exposure in genetically predisposed individuals, leading to damage of the small intestinal mucosa and impaired nutrient absorption [1,2]. The disease has a systemic character and may manifest with a wide spectrum of clinical symptoms, including gastrointestinal and extraintestinal manifestations, as well as oligosymptomatic or asymptomatic forms [1,3]. It is estimated that celiac disease affects approximately 1% of the general population, and its recognition has increased in recent decades [1,4].

The development of CD results from complex interactions between genetic, immunological, and environmental factors, with a key role played by HLA-DQ2 and HLA-DQ8 haplotypes [2,4]. Gluten exposure triggers an aberrant immune response leading to progressive villous atrophy, which constitutes the pathological hallmark of CD [1,2]. Diagnosis is based on serological testing and histopathological assessment of the small intestine, in accordance with current clinical guidelines [1,5,6].

Despite substantial advances in diagnostic methods, CD frequently remains undiagnosed for several years because of its heterogeneous and often nonspecific clinical presentation [1,3]. Delayed diagnosis may lead to metabolic, hematological, and skeletal complications and may also affect patients’ psychosocial functioning and quality of life [1,4]. Lifelong adherence to a gluten-free diet (GFD) remains the only effective treatment for CD, aiming to achieve symptom resolution, regeneration of the small intestinal mucosa, and prevention of disease-related complications [1,7]. Despite its effectiveness, maintaining a strict GFD constitutes a significant challenge, requiring constant monitoring of food intake and adaptation in everyday and social life [7]. Increasing attention has recently been devoted to the psychosocial consequences of CD, emphasizing that successful adaptation depends not only on clinical factors but also on cognitive and emotional processes involved in coping with chronic illness [8].

To better understand why some patients experience significant emotional distress and burden, while others adapt relatively well, theoretical models addressing cognitive vulnerability to depression provide a useful explanatory framework. According to cognitive vulnerability models, stable negative self-belief increases individuals’ sensitivity to stressful life events, which may lead to stronger emotional reactions and a higher risk of developing depressive symptoms in the context of chronic illness [9,10]. One well-established framework for understanding this cognitive vulnerability is Beck’s cognitive triad, which specifies negative views of the self, the world, and the future as central components of depressive schemas [11,12]. According to Riskind and Alloy [12], self-related beliefs are established early in life and constitute relatively stable cognitive structures that influence emotional responses to stressful life events, including chronic illness. Within cognitive vulnerability–stress models, including that proposed by Hilsman and Garber [10], stressful life events activate latent dysfunctional beliefs, increasing the likelihood of depressive symptoms.

A systematic review and meta-analysis have shown that individuals with CD have a significantly elevated risk of depression compared with healthy controls [13]. Large population-based studies have further confirmed higher rates of depressive disorders among individuals with CD, while longitudinal evidence suggests that childhood diagnosis is associated with an increased risk of psychiatric disorders later in life [14,15]. Depressive symptoms are also commonly reported among adults with biopsy-confirmed CD [16].

Beyond the increased risk of depressive symptoms, CD imposes substantial psychosocial and practical burdens. Lifelong adherence to a strict GFD requires constant vigilance, careful meal planning, and adaptations in social situations, which may interfere with family life and social participation. Persistent gastrointestinal symptoms, fatigue, and changes in physical appearance, including weight fluctuations or dermatological manifestations, can further affect self-esteem. Collectively, these challenges may function as chronic stressors capable of activating maladaptive cognitive schemas and reinforcing depressive symptoms, particularly in psychologically vulnerable individuals. Recognizing the complex interaction between dietary demands, disease manifestations, and emotional well-being is crucial for understanding psychological adjustment in patients with CD [8,17,18].

To our knowledge, depressive cognitive schemas have not previously been investigated in adults with CD. Investigating depressive cognitive schemas in conjunction with depressive symptoms in patients with CD provides a novel approach to better understand the psychological impact of chronic illness and dietary management [19]. By examining how negative cognitive schemas interact with the practical and psychosocial burdens of living with CD—such as dietary restrictions, symptom severity, and changes in physical appearance—this study addresses a critical gap in the literature [19]. Findings from this research may inform clinical practice by highlighting the importance of psychological screening and further investigation of cognitive and emotional factors relevant to psychological adjustment to CD and treatment-related behaviors [19].

Building on this rationale, the primary aim of the present study was to assess depressive cognitive schemas and depressive symptom levels among adults with CD in comparison with healthy controls. Specifically, we examined whether adults with CD exhibit higher levels of depressive symptoms, assessed using the Beck Depression Inventory (BDI), and more negative depressive cognitive schemas, measured with the Cognitive Triad Inventory (CTI), than healthy controls. We also explored associations between cognitive schemas, depressive symptoms, clinical characteristics, and perceived disease burden, including the impact of CD symptoms and lifelong GFD on daily functioning, social participation, and emotional well-being.

## 2. Method

### 2.1. Study Design and Participants

This cross-sectional study compared 96 adults with CD and 96 healthy controls. Data was collected between May 2018 and September 2021. Participants with CD were recruited consecutively from patients treated at the Department and Clinic of Gastroenterology, Dietetics, and Internal Diseases at Poznan University of Medical Sciences, either during hospitalization or through the affiliated outpatient clinic.

Healthy control participants were recruited using a convenience sampling approach by the study author from her family environment and the local community. Both groups completed the study questionnaires and provided sociodemographic information. For participants with CD, additional clinical information was obtained from medical records and during their clinical assessment. The investigator (KK) was available to clarify any questions during questionnaire completion.

The two groups were equal in size but were not individually matched according to age or sex.

#### Sample Size Calculation

The required sample size was determined a priori based on the estimated size of the target population of individuals with celiac disease in Poland, derived from previously published epidemiological data [20]. Assuming a significance level of 0.05 and an acceptable margin of error of 10%, the minimum required sample size was calculated using the sample size formula described by Zasępa [21]. The calculation yielded a minimum of 96 participants with CD. The same sample size was adopted for the healthy control group. Accordingly, the final study sample comprised 96 participants with celiac disease and 96 healthy controls.

### 2.2. Measures and Variables

Both groups provided socio-demographic information, including age, sex, place of residence, marital status, education, and employment. For CD patients, additional clinical data were recorded, such as age at diagnosis, duration from initial symptoms to diagnosis, diagnostic procedures, family history of CD, comorbidities, and anthropometric measures (weight, height, and body mass index [BMI]).

Disease-related burden and adherence to a GFD were assessed using investigator-developed single-item measures. These items were designed to capture selected aspects of patients’ subjective experience of CD and included self-reported severity of CD symptoms, persistence of symptoms following gluten exposure, adherence to a GFD, frequency of intentional gluten consumption, perceived impact of GFD requirements on social, family, and professional life, perceived impact of CD symptoms and disease exacerbations on everyday functioning, and perceived psychological burden associated with CD symptoms and GFD requirements. The items were assessed on a 10-point scale, with item-specific response anchors. For symptom severity, impact, and psychological burden, higher scores indicated greater severity or burden. For adherence to a GFD, higher scores indicated greater adherence, whereas for intentional gluten consumption, higher scores indicated more frequent consumption. The investigator-developed items were used as individual indicators of perceived disease- and diet-related burden and were not combined into a total disease-burden score.

### 2.3. Inclusion and Exclusion Criteria

Adults aged 18–65 years of either sex who provided written informed consent were eligible. Participants in the CD group were required to have a documented diagnosis of CD in their medical records. Healthy controls were eligible if they had no previously diagnosed CD and no diagnosed chronic disease. No serological testing for CD was performed in the control group. Individuals with intellectual disabilities were excluded from both groups.

### 2.4. Ethical Considerations

The authors assert that all procedures contributing to this work comply with the ethical standards of the relevant national and institutional committees on human experimentation and with the Helsinki Declaration of 1975, as revised in 2013. All procedures involving human participants were approved by the Bioethics Committee of Poznan University of Medical Sciences (approval nos. 527/2018, 377/19, and 890/20).

All participants were provided with comprehensive information regarding the study objectives and assured of confidentiality prior to providing written informed consent.

### 2.5. Study Measures

The BDI and CTI, as standardized psychological assessment tools, were employed to evaluate the variables of interest. Participants completed both questionnaires independently, with assistance from the investigator provided if clarification was required. The combination of the BDI and CTI allows for a comprehensive examination of the relationship between depressive symptoms and cognitive schemas, which is central to the aims of this study.

The BDI is a self-report instrument designed to measure the frequency, severity, and variety of depressive symptoms [22,23]. In this study, the Polish adaptation developed by Parnowski and Jernajczyk [24] was used. The BDI serves as both a screening and an assessment tool, allowing quantitative evaluation of depressive symptom severity. The inventory consists of 21 multiple-choice items, each scored from 0 to 3, corresponding to specific symptoms of depression, including sadness, insomnia, fatigue, guilt, self-dislike, somatic complaints, loss of sexual interest, and suicidal thoughts. The maximum total score is 63, and participants report on their experiences during the preceding month. A total score above 9 may indicate clinically relevant depressive symptoms, although a clinical diagnosis requires further psychiatric evaluation. The original version demonstrates excellent psychometric properties, including high reliability and validity, and the Polish adaptation has shown comparable reliability and diagnostic utility [22,23,24,25].

The CTI [26], used in its Polish adaptation by Śliwerski [27], comprises 36 items organized into three subscales assessing views of the self, the world, and the future. Each subscale contains 10 scored items, whereas six items serve as validity (filter) items. Participants rate their agreement with each statement using a seven-point Likert scale, yielding a total score ranging from 30 to 210. Negatively keyed items are reverse scored, so that higher scores indicate more positive views of the self, world, and future, whereas lower scores indicate more negative views. In the Polish validation study, the CTI showed high internal consistency, with a Cronbach’s alpha coefficient of 0.95 for the total scale and 0.77–0.91 for the three subscales. The instrument also showed strong correlations with established measures of depression, including the BDI, and anxiety, supporting its construct validity [26,27].

### 2.6. Statistical Methods

Descriptive statistics for continuous variables included mean values, 95% confidence intervals (CIs), ranges, and standard deviations (SDs). For categorical variables, absolute frequencies and percentages were reported. Investigator-developed single-item measures of disease-related burden and adherence to a GFD were treated as individual variables and analyzed separately; no composite disease-burden score was calculated.

The distribution of continuous variables was assessed using the Shapiro–Wilk test. Between-group comparisons for quantitative variables were performed using the Mann–Whitney U test for two groups and the Kruskal–Wallis test, followed by the Dunn-Bonferroni post hoc test, for comparisons involving multiple groups. For the Mann–Whitney U tests, the effect size was expressed as Cohen’s d, calculated as d = 2r/sqrt(1 − r^2) (where r = |Z/(n1 + n2)|), with the Z-score computed using a continuity correction, following the PQStat user manual [28].

Spearman’s rank-order correlation coefficients (rS) were calculated to examine associations between quantitative variables. Associations between categorical variables were analyzed using the chi-square test of independence or the Fisher-Freeman-Halton exact test when appropriate. To account for multiple comparisons, Bonferroni correction was applied where appropriate to predefined families of related tests. The adjusted significance level was determined based on the number of comparisons within each family. The families were defined according to the specific research questions addressed by each set of correlation analyses, rather than by treating all statistical tests in the manuscript as a single family.

The primary analysis was the between-group comparisons of BDI and CTI scores between participants with CD and healthy controls. Correlation analyses examining associations between psychological measures and demographic, clinical, disease-related, and diet-related variables were considered exploratory.

Internal consistency was assessed using Cronbach’s alpha (α) based on the current study sample. Cronbach’s alpha was calculated for the BDI total score, the CTI total score, and each of the three CTI subscales (Self-view, World-view, and Future-view).

Sociodemographic variables were recorded for both groups and were considered as potential confounding factors in the analyses of BDI and CTI scores. To account for differences between the groups in age, sex, education, marital status, and employment, additional multivariable logistic regression analyses were performed. In these models, group membership (CD vs. healthy controls) was specified as the dependent variable, while BDI score, CTI total score, and each of the three CTI subscale scores (Self-view, World-view, and Future-view) were entered separately as predictors, together with age, sex, education, marital status, and employment status as covariates. Adjusted odds ratios (ORs), 95% CIs, and corresponding *p*-values were reported. The adjusted ORs represent the association between a one-unit increase in the respective BDI or CTI score and the odds of belonging to the CD group, while holding the other variables in the model constant. Potential complete or quasi-complete separation in the logistic regression models was assessed in R (version 4.5.1) using the ‘detectseparation’ package. No complete or quasi-complete separation was detected, and all model coefficients had finite maximum likelihood estimates. The sparse education category was retained in the model; therefore, the corresponding odds ratio was interpreted cautiously because of its wide 95% confidence interval.

The threshold for statistical significance was set at *p* < 0.05.

All statistical analyses were conducted using Statistica 13 (TIBCO Software Inc., Palo Alto, CA, USA) and PQStat v.2.0.0.001.

## 3. Results

### 3.1. Sociodemographic Characteristics

The CD and control groups did not differ significantly with respect to age or weekly working hours. Significant between-group differences were observed for sex, education, marital status, and employment status (see Table 1).

The proportion of females was higher in the CD group than in controls (78.1% vs. 57.3%, *p* < 0.002). Educational level differed significantly between groups (*p* = 0.009), with a greater proportion of university-educated participants among CD patients and more individuals with vocational education in the control group.

Marital status also differed between groups (*p* = 0.034), with married participants more frequently represented in the CD group, whereas divorced participants were more common in the control group. Employment status varied significantly (*p* = 0.003); receipt of disability pension was reported only among CD patients, while unemployment was more frequent in the control group.

No statistically significant differences were found regarding place of residence (*p* = 0.080). For further details, see Table 1.

### 3.2. Clinical Characteristics of Patients with Celiac Disease

The mean age at diagnosis was 33.6 (SD 13.4) years (range: 1–60), with a median of 32 years. Patients reported a mean duration of symptoms prior to diagnosis of 5.4 (SD 5.1) years (range: 0.5–24; median: 4), indicating a substantial diagnostic delay. Mean height and weight were 166.1 (SD 7.8) cm and 57.8 (SD 9.7) kg, respectively, corresponding to a mean BMI of 20.9 (SD 3.0) kg/m^2^.

Most patients were diagnosed using endoscopic and histopathological examination (51.0%), followed by laboratory testing (39.6%). A small proportion were diagnosed based on clinical interview and symptom assessment alone (2.1%), while other diagnostic approaches were reported by 6.3% of participants.

A family history of celiac disease was reported by 25% of patients. Comorbid conditions were present in 45.8% of the sample, including autoimmune diseases in 27.1% of participants (see Table 2).

### 3.3. Adherence to a Gluten-Free Diet and Disease-Related Burden in Patients with Celiac Disease

Patients reported moderate overall severity of CD symptoms (mean 5.3; SD 2.7) and moderate persistence of symptoms following gluten exposure (mean 4.4; SD 2.5). Adherence to a GFD was rated relatively high (mean 7.2; SD 2.8; median 8), while the frequency of intentional gluten consumption was low to moderate (mean 3.3; SD 2.4).

The perceived negative impact of GFD requirements on daily and social functioning was moderate across the assessed domains, including social activity (mean 4.6; SD 2.0), family life (mean 4.1; SD 2.2), and professional functioning (mean 4.6; SD 2.0). Patients also reported a moderate perceived impact of CD symptoms and disease exacerbations on everyday life, including the burden associated with healthcare use and work absence (mean 4.1; SD 2.5).

The perceived psychological burden associated with CD symptoms and GFD requirements was relatively low to moderate (mean 3.4; SD 2.2). Overall, these findings indicate that patients reported relatively good adherence to a GFD, alongside a moderate perceived burden related to symptoms, dietary requirements, and selected aspects of everyday functioning (for details, see Table 3).

### 3.4. Internal Consistency and Intercorrelations of the BDI and CTI

Internal consistency was high for the BDI total score and the CTI total score in the total sample and in both study groups (Table 4). The Self-view subscale also showed high internal consistency, whereas the Future-view subscale showed acceptable internal consistency. In contrast, the World-view subscale showed low internal consistency in the total sample and in both groups.

The three CTI subscales were significantly and substantially intercorrelated in both groups. Among participants with CD, correlations ranged from rS = 0.79 to rS = 0.86, whereas among healthy controls they ranged from rS = 0.60 to rS = 0.85 (all *p* < 0.001; Table 4).

### 3.5. Depressive Symptoms Assessed by BDI

The mean BDI score was higher in the CD group (8.4; SD = 7.9; 95% CI: 6.8–10.0; range: 0–29) than in healthy controls (6.6; SD = 8.0; 95% CI: 5.0–8.3; range: 0–34). The between-group difference was statistically significant (Mann–Whitney U = 3850.5, Z = 1.99, *p* = 0.046), with a small effect size (Cohen’s d = 0.29).

No significant differences were observed between groups in the distribution of BDI-defined depression severity (*p* = 0.82). In the CD group, 70.8% of participants reported no depressive symptoms, compared with 76.0% in the control group. Mild and moderate depressive symptoms were observed in 15.6% and 9.4% of CD patients, respectively, versus 14.6% and 6.3% of controls. Severe depression was rare, affecting 4.2% of CD patients and 3.1% of controls. Detailed data are presented in Table 5.

### 3.6. Cognitive Schemas Assessed by CTI

The overall CTI score did not differ significantly between patients with CD (mean 151.60; SD 21.31; 95% CI 147.3–155.9; range 102–191) and healthy controls (mean 150.15; SD 22.74; 95% CI 145.6–154.7; range 86–192; *p* = 0.908). Similarly, no significant group differences were observed for the individual subscales assessing self-view, World-view, and future-view (Self-view: 52.99; SD 9.84 vs. 51.80; SD 10.98, *p* = 0.623; World-view: 48.24; SD 6.07 vs. 46.73; SD 6.00, *p* = 0.074; Future-view: 50.38; SD 6.99 vs. 51.61; SD 8.28, *p* = 0.088).

In both groups, the highest mean scores were observed for Self-view, followed by Future-view and World-view. These descriptive differences should be interpreted with caution, as the present analyses focused on between-group comparisons and did not test differences among the three CTI subscales within groups. Detailed statistics are provided in Table 6.

### 3.7. Multivariable Analysis of BDI and CTI Scores

To examine whether the observed between-group differences in BDI and CTI scores remained after accounting for sociodemographic characteristics, multivariable logistic regression analyses were performed. Group membership (CD vs. healthy controls) was modeled as the dependent variable, while BDI and CTI scores were examined separately as predictors, with adjustment for age, sex, education, marital status, and employment status.

After adjustment, none of the examined psychological measures was significantly associated with group membership. The adjusted odds ratio for BDI score was 1.024 (95% CI: 0.984–1.066, *p* = 0.248). Similarly, no significant associations were observed for CTI Self-view (OR = 1.015, 95% CI: 0.986–1.045, *p* = 0.307), World-view (OR = 1.036, 95% CI: 0.985–1.090, *p* = 0.172), Future-view (OR = 0.988, 95% CI: 0.949–1.027, *p* = 0.533), or the total CTI score (OR = 1.004, 95% CI: 0.991–1.018, *p* = 0.520) (Table 7).

Thus, after adjustment for the specified sociodemographic characteristics, BDI and CTI scores were not independently associated with celiac disease group membership.

### 3.8. Psychological Correlates of Demographic and Clinical Data

In the CD group, BMI was positively correlated with CTI World-view (rS = 0.26, *p* = 0.012) and Future-view (rS = 0.28, *p* = 0.006). Both associations remained statistically significant after Bonferroni correction for the four BMI–CTI correlations (adjusted α = 0.0125). The correlations between BMI and CTI Self-view (rS = 0.22 *p* = 0.038) and CTI total score (rS = 0.26, *p* = 0.013) did not remain significant after correction. BDI scores were not significantly associated with working hours, age at diagnosis, duration of symptoms prior to diagnosis, or BMI (all *p* ≥ 0.574). No significant differences in BDI scores were observed according to family history of CD, comorbidities, or autoimmune comorbidities (all *p* ≥ 0.147). The remaining associations between CTI scores and the demographic and clinical characteristics examined were also not statistically significant (Table 8).

### 3.9. Psychological Correlates of Celiac Disease and Dietary Burden

After applying the Bonferroni correction, significant associations were observed only for the CTI Self-view subscale. Higher Self-view scores were associated with a lower perceived negative impact of GFD requirements on family life (rS = −0.29, *p* = 0.004) and a lower perceived impact of CD symptoms and disease exacerbations on everyday life (rS = −0.31, *p* = 0.002). These correlations remained statistically significant after correction for multiple comparisons (adjusted α = 0.0056). No other correlations between CTI scores, including the CTI total score and the World-view and Future-view subscales, and disease- or diet-related burden remained significant after Bonferroni correction. Similarly, none of the correlations between BDI total score and the burden variables remained significant after correction (Table 9).

### 3.10. Associations Between Depressive Symptoms and Cognitive Schemas

Spearman correlation analyses revealed significant negative associations between BDI scores and all CTI dimensions in both study groups. In both groups, CTI self-view scores were significantly associated with BDI scores (CD group: rS = −0.39, *p* < 0.001; healthy controls: rS = −0.60, *p* < 0.001). Additionally, BDI scores were significantly negatively associated with World-view (CD group: rS = −0.38, *p* < 0.001; healthy controls: rS = −0.36, *p* < 0.001) and Future-view scores (CD group: rS = −0.37, *p* < 0.001; healthy controls: rS = −0.45, *p* < 0.001). The total CTI score was significantly associated with BDI scores (CD group: rS = −0.40, *p* < 0.001; healthy controls: rS = −0.54, *p* < 0.001) (see Table 10). According to the CTI scoring procedure, higher scores indicate more positive views of the self, world, and future. Thus, higher levels of depressive symptoms were associated with less positive cognitive schemas across all three CTI dimensions in both groups. All observed associations remained statistically significant after Bonferroni correction for the four correlations examined within each group (adjusted α = 0.0125).

## 4. Discussion

As indicated above, the assessment of mood disturbances in patients with CD in the present study was extended to include cognitive schemas related to the self, the world, and the future, which have not previously been examined in this clinical population. These dimensions were assessed using the CTI, which operationalizes the core components of the cognitive triad associated with depression. Overall, patients with CD exhibited slightly higher depressive symptom scores on the BDI compared with healthy controls, with a small effect size, whereas total and subscale scores on the CTI did not differ significantly between groups. In addition to the primary between-group comparisons, exploratory multivariable logistic regression models were used to examine whether BDI and CTI scores were associated with group membership after adjustment for sociodemographic differences between the groups. After adjustment for sociodemographic characteristics, neither BDI nor CTI scores were independently associated with group membership. These findings suggest that although depressive symptoms were modestly elevated in the CD group, the present study did not demonstrate group differences in cognitive schemas as assessed by the CTI.

From a clinical perspective, the mean duration of symptoms before CD diagnosis in our sample was 5.4 ± 5.1 years, consistent with the often heterogeneous presentation of CD in adults, including oligosymptomatic and extraintestinal manifestations [1,2]. In the present study, however, duration of symptoms before diagnosis was not significantly associated with depressive symptoms or CTI scores. As this was an exploratory analysis, these findings do not support diagnostic delay as a psychological risk marker in the present sample. Rather, the duration of symptoms before diagnosis is reported here as a descriptive clinical characteristic of the study population.

The high prevalence of comorbidities, including autoimmune disorders, is consistent with the clinical profile of CD and illustrates the complexity of the clinical characteristics of this patient population. Coexisting autoimmune conditions may be associated with greater treatment burden and healthcare utilization; however, in the present study, comorbidities were not significantly associated with depressive symptoms or cognitive schemas. As these associations were examined exploratorily, the absence of significant findings does not support comorbidities as psychological risk factors in the present sample. Rather, these clinical characteristics are reported as part of the broader clinical profile of the study population and should not be interpreted as explanatory factors for psychological burden based on the present findings [2,4].

The association between Self-view scores and perceived impact of symptoms and disease exacerbations on everyday life may reflect the way in which patients perceive and report the consequences of their illness. Persistent or severe symptoms after gluten exposure may be related to inadequate dietary control, cross-contamination, or coexisting gastrointestinal disorders such as irritable bowel syndrome, lactose intolerance, or small intestinal bacterial overgrowth. In some cases, frequent symptom exacerbations may also be observed in the context of complications or rare forms such as refractory CD, requiring objective clinical verification [1,2,6]. These possible clinical explanations, however, were not directly assessed in the present study and therefore cannot be considered mechanisms underlying the observed associations.

According to the conceptual framework proposed by Riskind and Alloy [12] (2006), self-related beliefs constitute a stable component of cognitive vulnerability, shaped throughout childhood, and remain relatively persistent across the lifespan. These beliefs influence emotional reactivity and information processing in response to stressful or threatening events, including the diagnosis of a chronic illness, potentially contributing to maladaptive emotional responses.

Cognitive theories remain among the leading explanatory models of depressive disorders. Individuals vulnerable to depression are characterized by maladaptive self-referential beliefs and negative cognitive styles that shape the interpretation of life events [12]. Cognitive distortions facilitate the assimilation of experiences into pre-existing schemas, which manifest as the negative cognitive triad comprising adverse views of the self, the world, and the future. In Beck’s [11] model, dysfunctional cognitive schemas are not only associated with depression but are considered causal mechanisms underlying its development, with the cognitive triad representing a core symptomatic expression alongside emotional and motivational disturbances. Thus, Beck’s [11] theory provides not only a descriptive account of depressive functioning but also a coherent explanatory framework for the development of depressive disorders, which has subsequently been expanded in cognitive vulnerability models [12].

Furthermore, the cognitive vulnerability–stress model proposed by Hilsman and Garber [10] assumes that exposure to critical life events may precipitate depressive symptoms primarily in individuals characterized by negative beliefs about themselves, their future, and the causes of adverse events. Within this framework, the diagnosis of a chronic disease and the necessity of long-term dietary management may represent persistent stressors that could be relevant to the expression of cognitive vulnerability. However, the present cross-sectional study does not allow us to determine whether disease-related stressors contribute to changes in cognitive schemas or whether pre-existing cognitive characteristics influence the perception of disease-related stress.

Previous studies have examined cognitive schemas in patients with depressive disorders [11,12]. Among clinical populations, Lefebvre [28] demonstrated that individuals with depression, both with and without chronic back pain, exhibit more pronounced cognitive distortions compared with non-depressed individuals, regardless of pain status. Moreover, patients with comorbid depression and chronic pain showed higher levels of catastrophizing, overgeneralization, and selective attention to negative stimuli compared with those without persistent pain [29].

To date, depressive cognitive schemas have not been systematically investigated in patients with CD. In addition to assessing cognitive schemas, this study evaluated patients’ perceived burden related to CD and adherence to a GFD. Patients reported moderate impacts on family life, social activity, and everyday life, including healthcare use and work absence. These self-reported challenges provide a novel perspective on the psychosocial demands of CD and serve as a context for examining the relationships between depressive symptoms, cognitive schemas, and subjective disease burden.

In the present study, exploratory bivariate analyses identified significant associations between CTI Self-view scores and two indicators of perceived disease- and diet-related burden: the perceived negative impact of GFD requirements on family life and the perceived impact of CD symptoms and disease exacerbations on everyday life. Higher Self-view scores, indicating more positive self-related beliefs, were associated with lower perceived burden in these domains. These associations remained statistically significant after Bonferroni correction. These findings indicate an association between self-related cognitive schemas and patients’ subjective perceptions of selected psychosocial and practical consequences of CD. However, because the analyses were cross-sectional and based on self-report measures, these associations should not be interpreted as evidence that self-related cognitive schemas determine or reduce perceived disease burden. Moreover, the observed associations should be interpreted in the context of the broader pattern of findings: BDI scores were significantly associated with all CTI dimensions in both study groups, indicating substantial overlap between depressive symptoms and cognitive schemas. Thus, the present findings do not establish that Self-view represents a uniquely or causally important cognitive factor in CD.

At the same time, CTI and BDI scores were largely independent of clinical variables, with no significant associations observed across the clinical characteristics examined. This pattern does not provide evidence that diagnostic delay or comorbidities were associated with psychological functioning in the present sample. Rather, the findings indicate that the psychological measures examined here showed relatively limited associations with the available clinical characteristics. Future studies could explore potential psychological and behavioral factors associated with depressive symptoms and cognitive schemas in patients with CD, such as anxiety levels, body image concerns, or alexithymia. Examining these factors longitudinally may help clarify the relationships among psychological characteristics, disease-related experiences, and perceived burden.

### 4.1. Implications for Clinical Practice

From a medical standpoint, the results highlight the importance of integrating psychological assessment with objective indicators of disease activity, including serological markers, nutritional status, and, when indicated, histological evaluation. Such an approach allows differentiation between disease activity and subjective perception of disease burden, which may contribute to more comprehensive individualized patient management [26]. These findings are consistent with an interdisciplinary approach to care involving gastroenterologists, dietitians, and mental health professionals. Brief psychological screening may be considered as part of routine follow-up to identify patients who report elevated depressive symptoms or substantial subjective disease burden, thereby facilitating appropriate psychological support [1,2,4,6].

The observed associations between self-view and selected indicato_rs_ of perceived disease-related burden also suggest that self-related cognitive processes may be relevant to the psychological experience of CD. However, because these associations were cross-sectional and did not demonstrate an association with GFD adherence, the present findings do not support the conclusion that interventions targeting cognitive schemas would improve dietary adherence. Cognitive-behavioral or schema-focused approaches may nevertheless be considered as potential approaches to address depressive symptoms, maladaptive self-perceptions, or perceived psychological burden in appropriately selected patients, with their effectiveness requiring evaluation in longitudinal or intervention studies.

### 4.2. Study Limitations

Several limitations of the present study should be acknowledged. First, the CD group included a higher proportion of women than the healthy control group. In addition, the groups differed with respect to educational attainment, marital status, and employment status. These sociodemographic differences may represent potential sources of confounding, particularly when interpreting between-group differences in depressive symptoms and other psychological outcomes. To address this issue, additional multivariable analyses were performed with adjustment for age, sex, education, marital status, and employment. Although the adjusted analyses did not demonstrate independent associations between BDI or CTI scores and group membership, residual confounding cannot be completely excluded. Furthermore, the a priori sample size calculation was based on the target population size and the assumed margin of error rather than on the statistical power required to detect specific differences in psychological outcomes. Therefore, adequate statistical power to detect small between-group effects cannot be assumed, and the nonsignificant findings from the adjusted analyses should be interpreted cautiously. The predominance of women may also limit the generalizability of the findings to male patients, particularly given documented sex-related differences in depressive symptomatology and cognitive processing styles. Future studies should therefore aim to include more socio-demographically balanced samples.

Second, the cross-sectional design of the study precludes any causal inferences regarding the directionality of the relationships between cognitive schemas, depressive symptoms, and disease-related burden. While significant associations were identified, the cross-sectional design does not allow us to determine whether less positive self-related schemas contribute to increased emotional burden and perceived disease impact, or whether prolonged exposure to disease-related stressors leads to the development or reinforcement of maladaptive cognitive patterns. Furthermore, the cross-sectional design does not allow assessment of how cognitive schemas and depressive symptoms may change over time or in response to psychological or other interventions. Longitudinal studies are therefore needed to clarify temporal and potentially bidirectional relationships among these variables and to examine their stability and changes over time, including in response to interventions.

Third, although basic clinical characteristics were collected, including comorbidities, the assessment of psychological functioning and perceived disease- and diet-related burden relied primarily on self-report measures. In particular, depressive symptoms, cognitive schemas, and disease-related burden were assessed using self-report questionnaires, which may be susceptible to reporting and response bias. In addition, disease-related burden and adherence to a gluten-free diet were assessed using investigator-developed single-item measures that had not undergone formal psychometric validation. These items were intended to capture specific and clinically relevant aspects of patients’ subjective experience of CD and dietary management rather than to constitute a validated multidimensional disease-burden scale. Furthermore, the CTI subscales showed variable internal consistency in the present sample, with particularly low reliability for the World-view subscale, and substantial intercorrelations among the three domains, warranting caution when interpreting the individual subscale scores.

The psychometric properties of the investigator-developed single-item measures of disease-related burden and dietary adherence have not been established, and findings based on these measures should therefore be interpreted as descriptive and exploratory. Moreover, because the principal psychological and disease-burden variables were assessed using self-report methods, the observed associations between CTI dimensions and perceived disease-related burden may have been influenced by shared method variance or general negative affect. Although the possibility of residual confounding and common-method effects cannot be excluded, these associations are interpreted as exploratory. The study did not include objective indicators of current disease activity (e.g., serological markers or histopathological data) or clinician-rated or structured psychiatric assessments. Therefore, future studies combining self-report instruments with objective clinical parameters and structured diagnostic interviews and validated disease-specific measures of disease burden and dietary adherence would allow for a more comprehensive evaluation of the relationships between somatic and psychological factors.

Fourth, the voluntary nature of participation and recruitment during routine clinical visits may have introduced self-selection bias. Individuals who were more engaged in their healthcare or more concerned about their psychological well-being may have been more likely to participate. Consequently, the study sample may not fully represent the broader population of individuals with CD.

And fifth, the study focused primarily on depressive symptoms and cognitive schemas and did not assess other potentially relevant psychological factors, such as anxiety, perceived stress, or coping strategies. These factors may also influence psychosocial burden and may interact with cognitive and emotional processes in individuals with celiac disease. Future studies should therefore consider a broader range of psychological variables to provide a more comprehensive understanding of psychosocial functioning in this population.

## 5. Conclusions

The findings indicate that psychological functioning in adults with celiac disease involves both depressive symptoms and cognitive schemas, while the observed associations between CTI Self-view and selected indicators of perceived disease- and diet-related burden warrant further investigation. Although depressive symptom scores were slightly higher in patients with CD than in healthy controls, CTI scores did not differ significantly between groups. However, these associations should be interpreted as correlational and do not establish a causal contribution of self-view to disease burden.

The findings also did not demonstrate an association between CTI or BDI scores and self-reported adherence to a gluten-free diet. Further longitudinal and intervention studies are needed to determine whether psychological characteristics are associated with changes in perceived disease burden or treatment-related behaviors over time.

## Figures and Tables

**Table 1:** Socio-demographic characteristics of the study sample.

Characteristic	CD Patients (*n* = 96)	Healthy Controls (*n* = 96)	*p*-Value
Sex			**<0.002**
Female	75 (78.1%)	55 (57.3%)	
Male	21 (21.9%)	41 (42.7%)	
Age, years	39.7 SD 14.2 (18–65)	40.0 SD 14.5 (19–65)	0.802
Educational level			**0.009**
Elementary	3 (3.1%)	2 (2.1%)	
Vocational	3 (3.1%)	15 (15.6%)	
Secondary	36 (37.5%)	40 (41.7%)	
University	54 (56.3%)	39 (40.6%)	
Marital status			**0.034**
Single	30 (31.3%)	32 (33.3%)	
Married	59 (61.5%)	44 (45.8%)	
Widowed	3 (3.1%)	7 (7.3%)	
Divorced	4 (4.2%)	13 (13.5%)	
Place of residence			0.080
Rural	12 (12.5%)	12 (12.5%)	
City < 25,000	13 (13.5%)	27 (28.1%)	
City 25,000–200,000	18 (18.8%)	17 (17.7%)	
City > 200,000	53 (55.2%)	40 (41.7%)	
Employment status			**0.003**
Employed (full/part-time)	75 (78.1%)	76 (79.2%)	
Retired	4 (4.2%)	7 (7.3%)	
Disability pension	11 (11.5%)	0 (0%)	
Unemployed	5 (5.2%)	12 (12.5%)	
Student	1 (1.0%)	1 (1.0%)	
Working hours/week	30.3 SD 19.8 (0–75)	32.9 SD 19.0 (0–70)	0.190

Notes: celiac disease (CD); values are presented as *n* (%) or mean with SDs (range); *p*-values in bold indicate statistically significant differences (*p* < 0.05).

**Table 2:** Clinical characteristics of patients with CD.

Characteristic	Value
Age at diagnosis, years	33.6 SD 13.4 (1–60), median 32
Duration of symptoms prior to diagnosis, years	5.4 SD 5.1 (0.5–24), median 4
Height, cm	166.1 SD 7.8 (154–188), median 166
Weight, kg	57.8 SD 9.7 (41–92), median 56
Method of diagnosis	
Laboratory test	38 (39.6%)
Endoscopy and histopathology	49 (51.0%)
Medical interview and symptom analysis	3 (3.1%)
Other	6 (6.3%)
Family history of CD	24 (25%)
Comorbidities	44 (45.8%)
Including autoimmune disorders (ADs)	26 (27.1%)

Notes: celiac disease (CD); values are presented as mean with SDs (range) or *n* (%).

**Table 3:** Self-reported burden of CD and adherence to a GFD.

Domain	Mean (SD)	Range	Median
Severity of CD symptoms (1–10) *	5.3 (2.7)	1–10	5
Persistence of CD symptoms following gluten exposure (1–10) *	4.4 (2.5)	1–10	4
Self-reported adherence to a GFD (1–10) *	7.2 (2.8)	1–10	8
Frequency of intentional gluten consumption (1–10) **	3.3 (2.4)	1–10	3
Perceived negative impact of GFD requirements on social activity (1–10) ***	4.6 (2.0)	1–10	5
Perceived negative impact of GFD requirements on family life (1–10) ***	4.1 (2.2)	1–10	5
Perceived negative impact of GFD requirements on professional life (1–10) ***	4.6 (2.0)	1–10	5
Perceived impact of CD symptoms and disease exacerbations on everyday life (1–10) ***	4.1 (2.5)	1–10	4
Perceived psychological burden of CD symptoms and GFD requirements (1–10) ***	3.4 (2.2)	1–9	3

Notes: celiac disease (CD); gluten-free diet (GFD). All items were rated on a 1–10 scale. For items marked with *, 1 indicated a low level of symptom severity, symptom persistence, or adherence to a GFD; 5 indicated a moderate level; and 10 indicated a very high level. For the item marked with **, 1 indicated never, 5 indicated once every few weeks, and 10 indicated at least once a week. For items marked with ***, 1 indicated that GFD requirements or CD symptoms had little negative impact, 5 indicated a moderate negative impact, and 10 indicated a very high negative impact. Values are presented as mean (SD), range, and median.

**Table 4:** Internal consistency of the BDI and CTI and intercorrelations among CTI subscales.

Measure	Total Sample, α (95% CI)	CD, α (95% CI)	Healthy Controls, α (95% CI)
BDI total	0.914 (0.896–0.931)	0.908 (0.879–0.932)	0.923 (0.899–0.944)
CTI total	0.911 (0.891–0.928)	0.910 (0.882–0.934)	0.914 (0.887–0.937)
CTI Self-view	0.893 (0.869–0.914)	0.901 (0.869–0.928)	0.892 (0.856–0.921)
CTI World-view	0.501 (0.388–0.601)	0.546 (0.398–0.670)	0.445 (0.261–0.598)
CTI Future-view	0.762 (0.708–0.809)	0.714 (0.621–0.792)	0.800 (0.735–0.855)
CTI subscale intercorrelations		rS	rS
Self-view − World-view, rS	—	**0.86**	**0.60**
Self-view − Future-view, rS	—	**0.84**	**0.85**
World-view − Future-view, rS	—	**0.79**	**0.66**

Notes. α = Cronbach’s alpha; CI = confidence interval; BDI = Beck Depression Inventory; CTI = Cognitive Triad Inventory; CD = celiac disease; rS = Spearman’s rank-order correlation coefficient; *p*-values in bold indicate *p* < 0.001.

**Table 5:** BDI scores and depression severity categories in the study groups.

Group	Mean (SD)	95% CI	Range	No Depression *n* (%)	Mild *n* (%)	Moderate *n* (%)	Severe *n* (%)	*p*-Value	*p*-Value (Depression Severity Distribution)
CD patients	8.4 (7.9)	6.8–10.0	0–29	68 (70.8%)	15 (15.6%)	9 (9.4%)	4 (4.2%)	**0.046**	0.82
Healthy controls	6.6 (8.0)	5.0–8.3	0–34	73 (76.0%)	14 (14.6%)	6 (6.3%)	3 (3.1%)		

Notes: Beck Depression Inventory (BDI); celiac disease (CD); values are presented as mean and SDs (range) and 95% confidence intervals (95% CI) or *n* (%); *p*-values in bold indicate statistically significant differences (*p* < 0.05).

**Table 6:** CTI subscale and total scores by study group.

Subscale	Group	Mean (SD)	95% CI	Range	*p*-Value
Self-view	CD group	52.99 (9.84)	51.0–55.0	25–69	0.623
	Healthy controls	51.80 (10.98)	49.6–54.0	22–70	
World-view	CD group	48.24 (6.07)	47.0–49.5	32–64	0.074
	Healthy controls	46.73 (6.00)	45.5–47.9	29–58	
Future-view	CD group	50.38 (6.99)	49.0–51.8	30–66	0.088
	Healthy controls	51.61 (8.28)	50.0–53.3	27–64	
Total score	CD group	151.60 (21.31)	147.3–155.9	102–191	0.908
	Healthy controls	150.15 (22.74)	145.6–154.7	86–192	

Note: Cognitive Triad Inventory (CTI); celiac disease (CD); values are presented as mean (SD), 95% confidence interval (95% CI), or range; *p*-values were obtained using the Mann–Whitney U test.

**Table 7:** Multivariable logistic regression analyses of BDI and CTI scores.

Psychological Measure	Adjusted OR	95% CI	*p*-Value
BDI total score	1.024	0.984–1.066	0.248
CTI Self-view	1.015	0.986–1.045	0.307
CTI World-view	1.036	0.985–1.090	0.172
CTI Future-view	0.988	0.949–1.027	0.533
CTI total score	1.004	0.991–1.018	0.520

Notes: Multivariable logistic regression models included group membership (celiac disease vs. healthy controls) as the dependent variable. Each psychological measure was entered separately as the main predictor, with adjustment for age, sex, education, marital status, and employment status. ORs represent the association between a one-unit increase in the respective psychological measure and the odds of belonging to the CD group. OR > 1 indicates higher odds of belonging to the celiac disease group, whereas OR < 1 indicates lower odds. Statistical significance was set at *p* < 0.05.

**Table 8:** Associations between BDI, CTI scores and CD patient characteristics.

Characteristics	CTI Self-View rS (*p*)	CTI World-View rS (*p*)	CTI Future-View rS (*p*)	CTI Total rS (*p*)	BDI Total rS (*p*)
Working hours/week	−0.04 (0.700)	−0.11 (0.305)	−0.03 (0.762)	−0.03 (0.499)	−0.06 (0.574)
Age at diagnosis, years	−0.01 (0.936)	0.01 (0.959)	−0.06 (0.579)	−0.02 (0.843)	−0.02 (0.845)
Duration of symptoms prior to diagnosis, years	−0.04 (0.684)	−0.06 (0.577)	−0.07 (0.494)	−0.09 (0.399)	−0.01 (0.913)
BMI	0.22 (0.038)	**0.26 (0.012)**	**0.28 (0.006)**	0.26 (0.013)	−0.03 (0.762)
Characteristics	*p*	*p*	*p*	*p*	
Family history of CD	0.463	0.831	0.230	0.390	0.147
Comorbidities	0.600	0.464	0.290	0.337	0.699
Autoimmune comorbidities	0.108	0.381	0.536	0.322	0.753

Note: Cognitive Triad Inventory (CTI); Beck Depression Inventory (BDI); celiac disease (CD); Body Mass Index (BMI); rS = Spearman rank correlation coefficient. For continuous characteristics, Spearman rank correlation coefficients and *p*-values are reported; for categorical characteristics, *p*-values from the corresponding group-comparison tests are reported. Bonferroni correction was applied where appropriate; for the four BMI–CTI correlations, the adjusted significance level was α = 0.0125. Bold values indicate statistically significant results after the applicable correction.

**Table 9:** Spearman correlations between disease- and diet-related variables and CTI and BDI.

Disease-/Diet-Related Variables	CTI Self-View rS (*p*)	CTI World-View rS (*p*)	CTI Future-View rS (*p*)	CTI Total rS (*p*)	BDI Total rS (*p*)
Severity of CD symptoms (1–10)	−0.08 (0.422)	−0.08 (0.451)	−0.05 (0.603)	−0.08 (0.411)	0.10 (0.336)
Persistence of CD symptoms following gluten exposure (1–10)	−0.10 (0.313)	−0.04 (0.732)	−0.04 (0.691)	−0.09 (0.382)	0.10 (0.313)
Self-reported adherence to a GFD (1–10)	0.23 (0.027)	0.19 (0.066)	0.15 (0.151)	0.20 (0.048)	−0.14 (0.172)
Frequency of intentional gluten consumption (1–10)	−0.16 (0.117)	−0.17 (0.106)	−0.12 (0.258)	−0.13 (0.192)	0.02 (0.838)
Perceived negative impact of GFD requirements on social activity (1–10)	−0.10 (0.354)	−0.15 (0.147)	−0.07 (0.502)	−0.08 (0.417)	0.14 (0.163)
Perceived negative impact of GFD requirements on family life (1–10)	**−0.29 (0.004)**	−0.25 (0.014)	−0.21 (0.044)	−0.24 (0.020)	0.14 (0.183)
Perceived negative impact of GFD requirements on professional life (1–10)	−0.09 (0.364)	−0.15 (0.143)	−0.05 (0.641)	−0.07 (0.489)	0.16 (0.112)
Perceived impact of CD symptoms and disease exacerbations on everyday life (1–10)	**−0.31 (0.002)**	−0.18 (0.074)	−0.24 (0.020)	−0.26 (0.011)	0.16 (0.109)
Perceived psychological burden of CD symptoms and GFD requirements (1–10)	−0.28 (0.006)	−0.21 (0.045)	−0.13 (0.191)	−0.23 (0.024)	0.25 (0.013)

Note: Cognitive Triad Inventory (CTI); Beck Depression Inventory (BDI); celiac disease (CD); gluten-free diet (GFD); rS = Spearman rank correlation coefficient; bold values indicate statistically significant after Bonferroni correction (adjusted α = 0.0056).

**Table 10:** Spearman correlations between BDI and CTI scores in both study groups.

CTI Subscale/Total	CD Group: rS	*p*-Value	Healthy Controls: rS	*p*-Value
Self-view	**−0.39**	**<0.001**	**−0.60**	**<0.001**
World-view	**−0.38**	**<0.001**	**−0.36**	**<0.001**
Future-view	**−0.37**	**<0.001**	**−0.45**	**<0.001**
CTI total score	**−0.40**	**<0.001**	**−0.54**	**<0.001**

Note: Cognitive Triad Inventory (CTI); Beck Depression Inventory (BDI); celiac disease (CD); rS = Spearman rank correlation coefficient; bold values statistically significant after Bonferroni correction (adjusted α = 0.0125).

## Data Availability

The data presented in this study are available on request from the corresponding author. The data are not publicly available due to privacy.
